# Methylome and transcriptome analyses of apple fruit somatic mutations reveal the difference of red phenotype

**DOI:** 10.1186/s12864-019-5499-2

**Published:** 2019-02-07

**Authors:** Sheng-Hui Jiang, Qing-Guo Sun, Min Chen, Nan Wang, Hai-Feng Xu, Hong-Cheng Fang, Yi-Cheng Wang, Zong-Ying Zhang, Xue-Sen Chen

**Affiliations:** 1 College of Horticulture Science and Engineering, Shandong Agricultural University, 61 Daizong Road, Tai’an, 271018 China; 2 State Key Laboratory of Crop Biology, Shandong Agricultural University, 61 Daizong Road, Tai’an, 271018 China; 3 Collaborative Innovation Center of Fruit & Vegetable Quality and Efficient Production in Shandong, 61 Daizong Road, Tai’an, 271018 China

**Keywords:** Apple, Red mutant, Anthocyanin, Methylomes, Transcriptomes, Methylation, Gene expression

## Abstract

**Background:**

Fruit peel colour is an important agronomic trait for fruit quality. Cytosine methylation plays an important role in gene regulation. Although the DNA methylation level of a single gene is important to affect the phenotype of mutation, there are large unknown of difference of the DNA methylation in plant and its mutants.

**Results:**

Using bisulfite sequencing (BS-Seq) and RNA-sequencing (RNA-Seq), we analysed three deep-red-skinned apple (*Malus* × *domestica*) mutants (Yanfu 3, YF3; Yanfu 8, YF8; Shannonghong, SNH) and their lighter-skinned parents (Nagafu 2, NF2; Yanfu 3, YF3; Ralls, RL) to explore the different changes in methylation patterns associated with anthocyanin concentrations. We identified 13,405, 13,384, and 10,925 differentially methylated regions (DMRs) and 1987, 956, and 1180 differentially expressed genes (DEGs) in the NF2/YF3, YF3/YF8, and RL/SNH comparisons, respectively. And we found two DMR-associated DEGs involved in the anthocyanin pathway: *ANS* (MD06G1071600) and *F3H* (MD05G1074200). These genes exhibited upregulated expression in apple mutants, and differences were observed in the methylation patterns of their promoters. These results suggested that both the regulatory and structural genes may be modified by DNA methylation in the anthocyanin pathway. However, the methylation of structural genes was not the primary reason for expression-level changes. The expression of structural genes may be synergistically regulated by transcription factors and methylation changes. Additionally, the expression of the transcription factor gene *MYB114* (MD17G1261100) was upregulated in the deep-red-skinned apple.

**Conclusion:**

Through the analysis of global methylation and transcription, we did not find the correlation between gene expression and the DNA methylation. However, we observed that the upregulated expression of *ANS* (MD06G1071600) and *F3H* (MD05G1074200) in apple mutants results in increased anthocyanin contents. Moreover, MYB114 (MD17G1261100) is likely another regulatory gene involved in apple coloration. Our data provided a new understanding about the differences in formation of apple colour mutants.

**Electronic supplementary material:**

The online version of this article (10.1186/s12864-019-5499-2) contains supplementary material, which is available to authorized users.

## Background

Fruit peel colour is an important agronomic trait for fruit quality. The types and concentrations of anthocyanins determine the fruit colour of apple [1, 2], pear [3], and grape [4, 5]. The anthocyanin biosynthetic pathway has been elucidated in model plants [6], and also in apple. Structural and regulatory genes in the anthocyanin biosynthetic pathway have been isolated and identified. The MBW complex comprising the MYB, basic helix-loop-helix protein (bHLH), and WD40 protein regulates the transcriptional level of structural genes. MdMYB1 and MdMYBA, which regulate anthocyanin biosynthesis in apple peel, have been isolated and the expression of their encoding genes has been shown to be strongly induced by light [7, 8]. MdbHLH3 can bind to the promoters of *MdDFR* and *MdUFGT* and activate their expression, which promotes anthocyanin biosynthesis [9]. However, MdTTG1, a WD40 transcription factor, cannot interact with MdMYB1 and bind to the promoters of *MdDFR* and *MdUFGT*. Instead, MdTTG1 may regulate anthocyanin biosynthesis by interacting with MdbHLH3 and MdbHLH33 [10]. Anthocyanin biosynthesis in apple is also affected by light, temperature, and hormones. The transcriptional level of *MdMYBA* increases under low temperature, then MdMYBA binds to the promoter of *ANS* and promotes anthocyanin accumulation [8]. MdbHLH3 can be phosphorylated under low temperature to enhance its transcriptional activation activity, leading to increased anthocyanin biosynthesis [9]. Additionally, high temperature can prevent the colour formation in apple fruit through influencing the activity of the MBW complex [11]. Jasmonic acid can promote the accumulation of anthocyanins by enhancing the binding of MdMYB9 and MdMYB11 to the promoter of downstream structural genes [12]. Moreover, sucrose-induced anthocyanin accumulation is regulated by the interaction between MdSnRK1.1 and MdJAZ18 [13].

Cytosine methylation is widespread in eukaryotes and plays essential roles in various processes, including the maintenance of genome integrity, regulation of transcription, silencing of transposable elements, and imprinting [14]. In plants, cytosine methylation exists in three different sequence contexts: CG, CHG, and CHH (where H represents A, T, or C) [15]. In *Arabidopsis thaliana*, the following three types of DNA methyltransferases catalyse the transfer of the methyl group onto cytosine in the DNA to form 5-methylcytosine: METHYLTRANSFERASE 1 (MET1), CHROMOMETHYLASE 2 and 3 (CMT2 and CMT3), and DOMAINS REARRANGED METHYLTRANSFERASE 2 (DRM2). Specifically, MET1 and CMT3 maintain methylation in the CG and CHG sequence contexts, respectively [15], whereas DRM2 is responsible for maintaining the methylation of CHH sites [16]. To some extent, CMT2 is redundant with CMT3 in CHG methylation; instead, its main function is in CHH methylation together with DRM2 [17]. The chromatin remodelling protein, DECREASE IN DNA METHYLATION 1 (DDM1) is responsible for maintaining the methylation of all three sequence contexts after DNA replication [18]. Cytosine methylation can be erased in plants by some DNA glycosylases, including DEMETER (DME), REPRESSOR OF SILENCING 1 (ROS1), DEMETER-LIKE 2 (DML2) and DML3 [19–21].

In woody crop species, somatic mutations (bud sport or sport) are important for the discovery of new cultivars or strains with traits superior to those of the parents. For fruit trees, sport selection can improve agronomic traits such as fruit colour, plant type, and maturation time. About 30% of existing apple cultivars have been selected from sports, most of them red-fruited. Sport cultivars account for 50% of global apple yield [22]. The mechanisms of some somatic mutations have been studied in apple and pear. For example, the methylation level of the *MdMYB1* promoter was found to be lower in the red ‘Ralls’ sport than in ‘Ralls’ [23]. In the apple mutant ‘Blondee’ with an anthocyanin-deficient yellow skin, the methylation level of the *MdMYB10* promoter was higher than that in the parent ‘Kidd’s D-8’ [2]. In pear, the green-skinned sport of ‘Max Red Bartlett’ was found to have a higher methylation level of the *PcMYB10* promoter [3] and the pigmentation pattern of the red sport of ‘Zaosu’ pear was shown to be associated with demethylation of the *PyMYB10* promoter [24]. Thus, DNA methylation plays an important role in fruit colour mutations, but the differences in the DNA methylation of somatic mutants and their parents remain unclear.

In this study, we performed transcriptome and methylome analyses to different changes of colour variation in apple. We used deep-red-skinned apple mutants and their light-red-skinned parents as the experimental materials. Global methylation and transcription analyses indicated that structural genes (*ANS* and *DFR*) may be differentially methylated between differently coloured varieties. The expression levels of *ANS* and *DFR* were upregulated in deep-red-skinned apple, which affected the anthocyanin pathway. Furthermore, the expression of an R2R3-MYB gene, *MYB114,* was upregulated in two fully red apples, suggesting this gene may be involved in anthocyanin regulation during apple coloration. These findings brought us the new view to understand the different between apple and its colour mutants.

## Results

### Anthocyanin contents in five apple cultivars

**Fig. 1:**
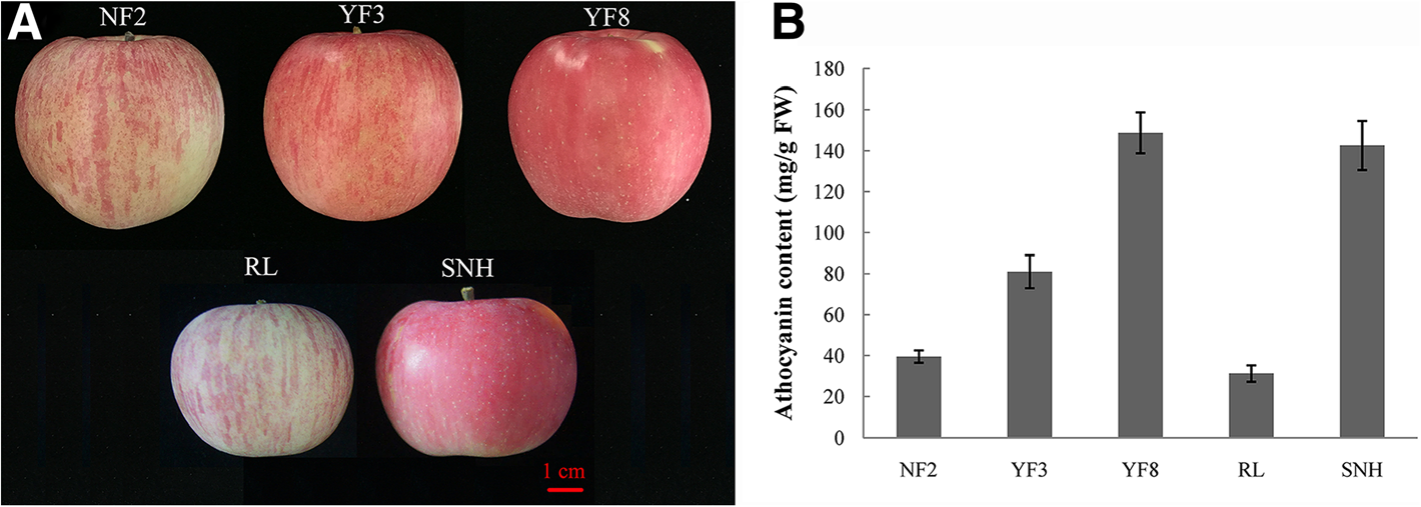
Phenotypes of five apple cultivars. **a** Fruit phenotypes of five apple cultivars. **b** Anthocyanin content in fruit skins of five apple cultivars. NF2, YF3, YF8, RL, and SNH represent Nagafu 2, Yanfu 3, Yanfu 8, Ralls, and Shannonghong, respectively. Red-fruit-skinned mutants are YF3, YF8, and SNH, and lighter-fruit-skinned parents are NF2, YF3 and RL

YF3 and YF8 are somatic mutants or bud sports that were selected from NF2 and YF3, respectively, and SNH, also a red sport cultivar, was selected from RL. The genetic fingerprinting data did not show any differences among NF2, YF3 and YF8. Additionally, there were no differences between RL and SNH, confirming the relationship between the parents and sports (Additional file 1: Figure S1). The three mutants produce deep-red-skinned fruit, whereas their parents produce lighter red fruit. Fruits of NF2, YF3, and RL exhibit a red-striped pattern, whereas those of YF8 and SNH are fully red (Fig. 1a). We measured the anthocyanin content in the fruit skins of the five cultivars; the mutants had markedly higher anthocyanin concentrations in the fruit skins (Fig. 1b). Thus, the red apple sports were selected because they contained higher anthocyanin contents than their parents.

### Bisulfite sequencing of genomes of different apple cultivars

According to recent reports, methylation of a single gene can explain fruit colour mutations in some fruit trees, including apple [1, 2, 23] and pear [3, 24]. On the basis of this information, we prepared three pairs of libraries (five cultivars) from different apple sports showing deep-red or light-red fruit skin colour. In the single-base DNA methylation BS-Seq analyses of the three groups of samples, about 250,000,000 clean reads were filtered from each sample, yielding a read depth about 38 × the new apple genome [25]. From the data, 162,443,704 to 217,667,799 mapped reads and 142,453,711 to 187,657,786 uniquely mapped reads were obtained (64.98–87.07% and 56.98–75.06%, respectively) (Additional file 2: Table. S1). The bisulfite conversion rates of the five cultivars NF2, YF3, YF8, RL, and SNH were 99.57, 99.58, 99.58, 99.40, and 99.43%, respectively (Additional file 2: Table. S1). The cumulative distribution of C-base effective sequencing depth is shown in Additional file 1: Figure S2a. The fraction of covered of three sequence contexts (CG, CHG and CHH) close to 100% with the minimum depth; there were peaks at approximately 10–20× read depth with additional peaks at the minimum read depth, and long tails (Additional file 1: Figure S2a). These results suggested that the sequencing generated high-quality data. The sequencing depth distribution for the five cultivars is presented in Additional file 1: Figure S2b. The relative proportions of methylcytosines (mCs) in three sequence contexts (CG, CHG and CHH) for the five apple cultivars were calculated (Additional file 1: Figure S2c), the mC frequency was highest at CHH sites (56–61%), and lower at CG and CHG sites (21–24% and 18–20%, respectively). The methylation landscapes were similar among the five apple genomes from a genome-wide perspective. The five apple genomes are presented in Additional file 1: Figure S3. The distribution of mCs in all sequence contexts on all 17 apple chromosomes is shown in Additional file 1: Figure S4.

### DNA methylation patterns in different apple genomic regions

**Fig. 2:**
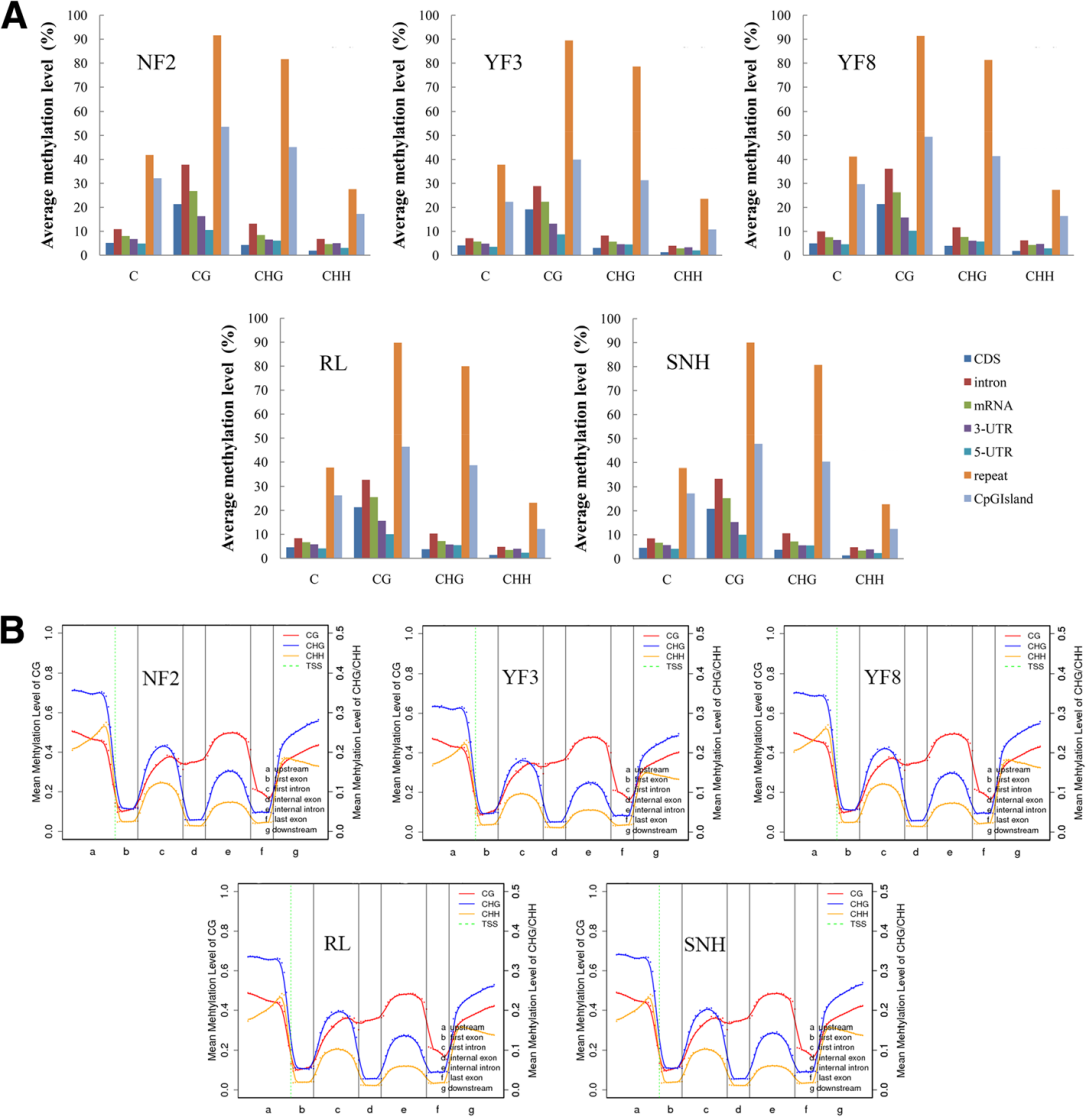
Average methylation levels of elements and entire transcriptional units. **a** Effective coverage of some types of elements. **b** DNA methylation patterns across entire transcriptional units at whole genome level. NF2, YF3, YF8, RL and SNH represent Nagafu 2, Yanfu 3, Yanfu 8, Ralls, and Shannonghong, respectively. The green dotted line between a and b indicates transcriptional start site (TSS)

To investigate the DNA methylation patterns in different apple genomic regions, we analysed the methylation profiles in gene regions (Additional file 2: Table. S2). The methylation level was highest in the CG context followed by CHG and then CHH in each gene region of the five apple cultivars (Fig. 2a). Repeat and CpG -island regions had the highest methylation levels of the different gene regions, suggesting that these two regions are epigenetic regulatory regions that may alter gene expression. The distribution of DNA methylation levels in various gene features are presented in Fig. 2b. For the CG context, CG methylation was more abundant in upstream and downstream regions than in exon regions, and methylation levels were much higher in introns than in exons. Methylation was more frequent in the CHG context than in the CG and CHH contexts in upstream, first intron, and downstream regions. Of the three contexts, the CHH context had the lowest methylation level in exons and introns (Fig. 2b).

### Differentially methylated regions among differently coloured apple cultivars

**Fig. 3:**
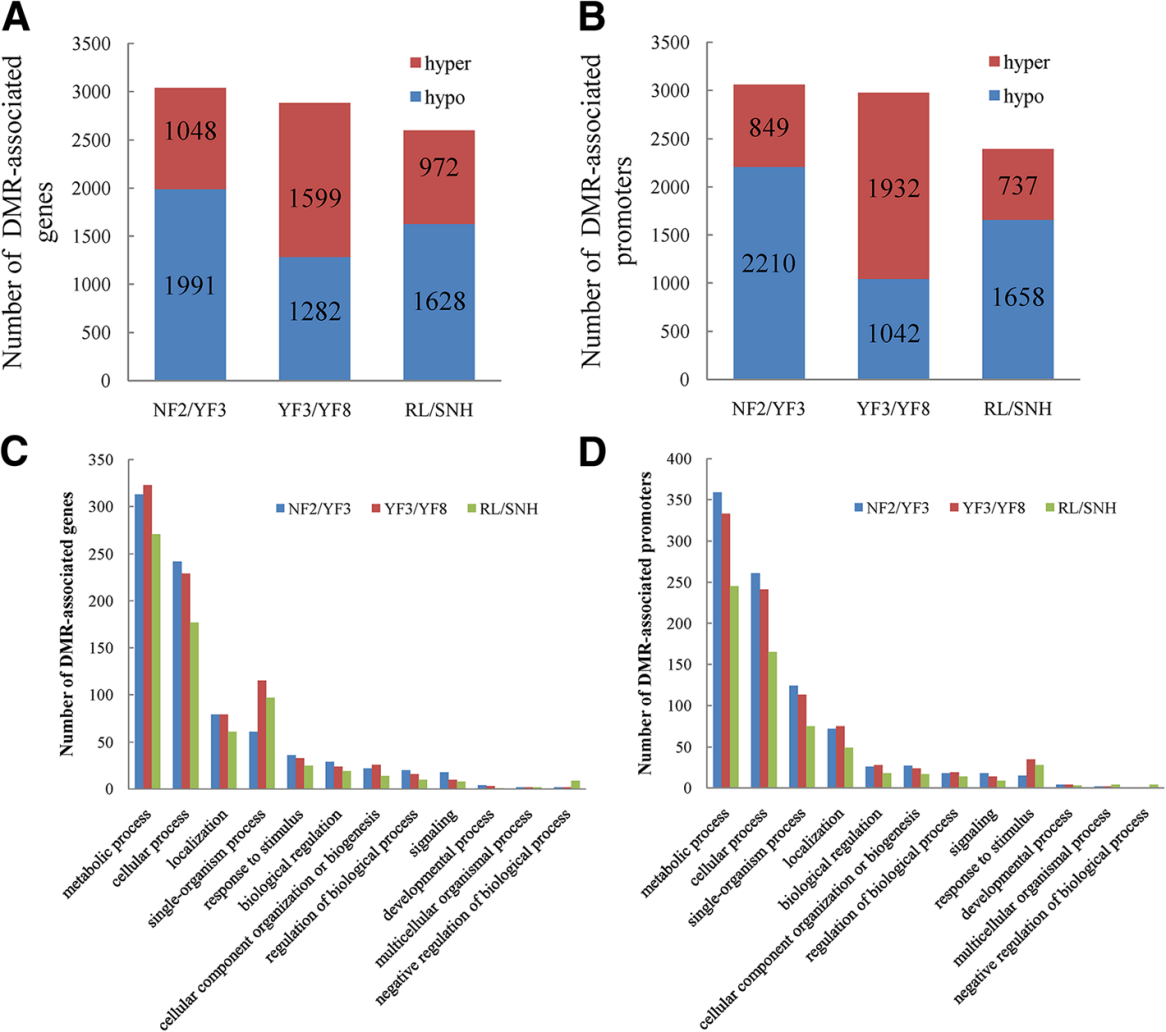
Differential methylation among apple cultivars. Number of differentially methylated regions (DMRs including hyper and hypo) -associated genes (**a**) and promoters (**b**) in NF2 and YF3 (NF2/YF3), YF3 and YF8 (YF3/YF8) and RL and SNH (RL/SNH) comparisons. Gene ontology (GO) categories significantly enriched in DMR-associated genes (**c**) and promoters (**d**) among three comparisons

To study the differential methylation among differently coloured apple mutants, we identified the DMRs. The number of DMRs on each chromosome and the length distribution of DMRs are listed in Additional file 2: Table S3. We detected 13, 405 DMRs between NF2 and YF3 (NF2/YF3), 13, 384 DMRs between YF3 and YF8 (YF3/YF8) and 10, 925 DMRs between RL and SNH (RL/SNH) (Additional file 2: Table S3). We divided the DMRs into the following two groups: DMR-associated genes and DMR-associated promoters, which DMRs overlapped with the genes and promoters, respectively. We identified 3039 DMR-associated genes in NF2/YF3, 2, 881 in YF3/YF8, and 2, 600 in RL/SNH (Fig. 3a). We found 3, 059 DMR-associated promoters in NF2/YF3, 2, 974 in YF3/YF8, and 2, 395 in RL/SNH (Fig. 3b). In NF2/YF3 and RL/SNH, there were more hypo DMR-associated genes and promoters than hyper DMR-associated genes and promoters; the opposite pattern was detected for YF3/YF8 (Fig. 3b). These results indicated that hypermethylation was more common in NF2 and RL than in their sport mutants, while hypermethylation was more common in YF8 than in its parent. A gene ontology (GO) analysis of DMR-associated genes indicated they were involved in diverse biological processes, including metabolic processes, cellular process, localization, single-organism process, and response to stimulus (Fig. 3c). The GO analysis of DMR-associated promoters was associated with genes involved in cellular process, localization, single-organism process, and biological process (Fig. 3d). The results of KEGG pathway analyses showed that the DMRs were enriched mostly in betalain biosynthesis and lipoic acid metabolism (Additional file 1: Figure S5).

### Differential gene expression among different cultivars

**Fig. 4:**
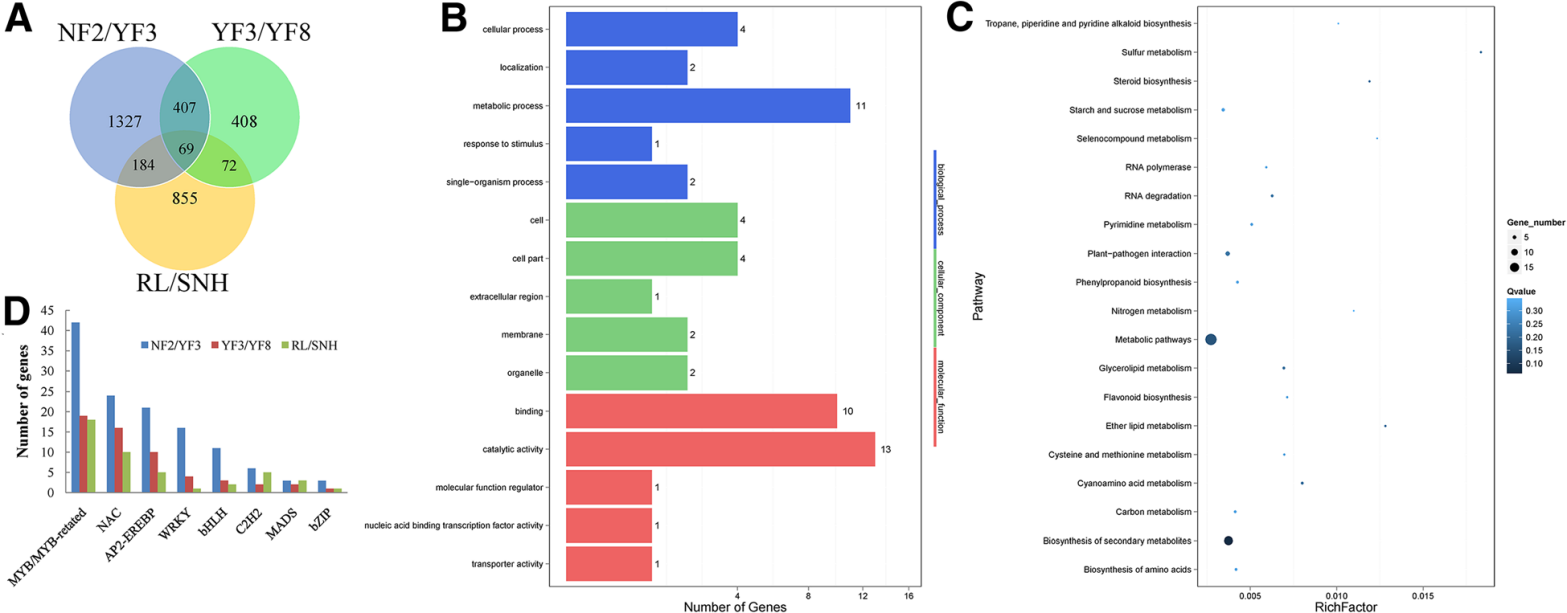
Differential gene expression among apple cultivars and GO enrichment analysis. **a** Venn diagram indicating the number of differentially expressed genes in NF2/YF3, YF3/YF8, and RL/SNH comparisons. **b** Results of a GO enrichment analysis of differentially expressed genes in NF2/YF3, YF3/YF8, and RL/SNH. **c** Results of a KEGG pathway analysis of differentially expressed genes in NF2/YF3, YF3/YF8, and RL/SNH comparisons. **d** Number of highly represented transcription factors among differentially expressed genes in NF2/YF3, YF3/YF8, and RL/SNH comparisons

We used an RNA-Seq approach to explore differential gene expression among the different cultivars. Specifically, 15 apple fruit peel samples (three from each of five cultivars) were used in the RNA-Seq analyses. The sequencing data are summarised in Additional file 2: Table S4. Transcripts with at least a 2-fold change in abundance and with *p* < 0.05 were regarded as differentially expressed genes (DEGs) (Additional file 1: Figure S6a, b, c). A total of 1987 (893 upregulated and 1094 downregulated), 956 (501 upregulated and 455 downregulated) and 1180 (714 upregulated and 466 downregulated) transcripts were differentially expressed in the NF2/YF3, YF3/YF8 and RL/SNH comparisons, respectively (Additional file 1: Figure S6d). In the GO analysis, the DEGs in these three groups were distributed into 37 functional terms as follows: 14–16 terms for biological process; 9–11 terms for cellular component, and 8–11 terms for molecular function. Most the biological process genes were involved in metabolic process, cellular process, and single organism process. The genes in the cellular component group were mainly related to cell, cell part, and organelle. Many genes in the molecular function group were related to binding and catalytic activity (Additional file 1: Figure S7a). The KEGG pathway enrichment analysis indicated that the DEGs were significantly enriched in the pathways of plant–pathogen interaction, as well as the metabolism and biosynthesis of secondary metabolites (Additional file 1: Figure S7b). Of these DEGs, 69 were differentially expressed in all three comparisons (Fig. 4a). The GO analysis indicated that these genes were involved in diverse biological processes and molecular functions, such as metabolic process, binding, and catalytic activity (Fig. 4b). An enrichment analysis of DEGs revealed that genes involved in metabolic processes and biosynthesis of secondary metabolites were significantly overrepresented (Fig. 4c). Additionally, at least 292 genes encoding transcription factors (TF) belonging to 32 families were identified as DEGs between the parents and their mutants. These genes belonged to the *MYB/MYB*-related, *NAC*, *AP2-EREBP*, *WRKY*, and *bHLH* families and were the most highly represented genes among the DEGs (Fig. 4d). Genes encoding C2H2, MADS, and bZIP TFs were also among the DEGs in the all three groups.

### Analysis of DEGs related to the anthocyanin pathway

**Fig. 5:**
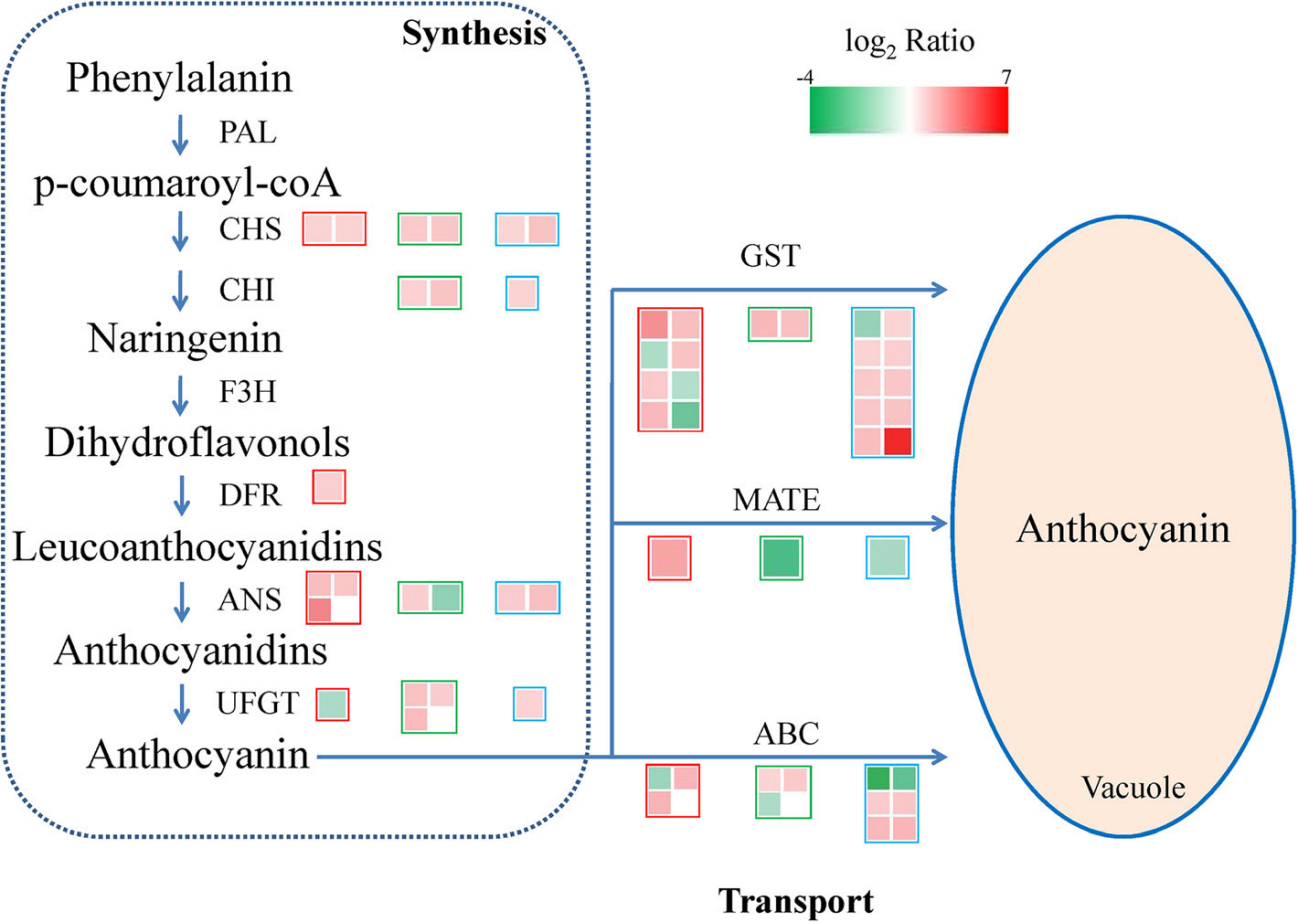
Expression patterns of several genes involved in anthocyanin pathway in NF2/YF3, YF3/YF8 and RL/SNH comparisons. Value of log_2_ (fold change) is distinguished by colour: green represents downregulation, and red represents upregulation. Red, green, and blue boxes represent the differentially expressed genes in NF2/YF3, YF3/YF8, and RL/SNH comparisons, respectively. The boxes on the same line represent the same gene family of the three comparisons. *CHS*, chalcone synthase; *CHI*, chalcone isomerase; *F3H*, flavanone 3-hydroxylase; *DFR*, dihydroflavonol 4-reductase; *ANS*, anthocyanidin synthase; *UFGT*, flavonoid-3-O-glucosyltransferase; *GST*, glutathione S-transferase; *ABC*, ATP-binding cassette; *MATE*, multidrug and toxic compound extrusion

To investigate the differential gene expression among differently coloured cultivars, we focused on DEGs involved in anthocyanin synthesis (Additional file 2: Table S5; Fig. 5). The upstream genes in the anthocyanin synthesis pathway include chalcone synthase (*CHS*),chalcone isomerase (*CHI*), and flavanone-3-hydroxylase (*F3H*), while the downstream genes include dihydroflavonol-4-reductase (*DFR*), leucoanthocyanidin dioxygenase (*ANS*), and UDP-glucose:flavonoid 3-O-glucosyltransferase (*UFGT*). The expression levels of two *CHS*, one *DFR*, and three *ANS* genes were upregulated, whereas the expression of one *UFGT* gene was downregulated in YF3 compared with NF2. In contrast, the expression levels of two *CHS*, two *CHI*, one *ANS,* and three *UFGT* genes were upregulated, whereas the expression of one ANS gene was downregulated in YF8 compared with YF3. These results implied that the upregulated expression of three genes (MD04G1003300, *CHS*; MD03G1001100, *ANS*; and MD17G1055500, *UFGT*) may have contributed to the increased anthocyanin synthesis in the three ‘Fuji’ cultivars. Additionally, the upregulated expression of two *CHS*, one *CHI*, two *ANS*, and one *UFGT* genes in SNH compared with RL was responsible for the greater anthocyanin synthesis in SNH.

We also analysed the genes encoding transporters in the anthocyanin pathway, including glutathione S-transferase (*GST*), multidrug and toxic compound extrusion (*MATE*) and the ATP-binding cassette (*ABC*). The expression levels of three *GST*, one *MATE,* and one *ABC* genes were upregulated in NF2/YF3. Meanwhile, the expression levels of two *GST* and two *ABC* genes were upregulated in YF3/YF8, whereas the expression levels of nine *GST* and three *ABC* genes were upregulated in RL/SNH. These results indicated that anthocyanin transport was enhanced in the red mutants compared with their lighter-skinned parents.

### Interconnection of DMRs and DEGs in differently coloured apple lines

**Fig. 6:**
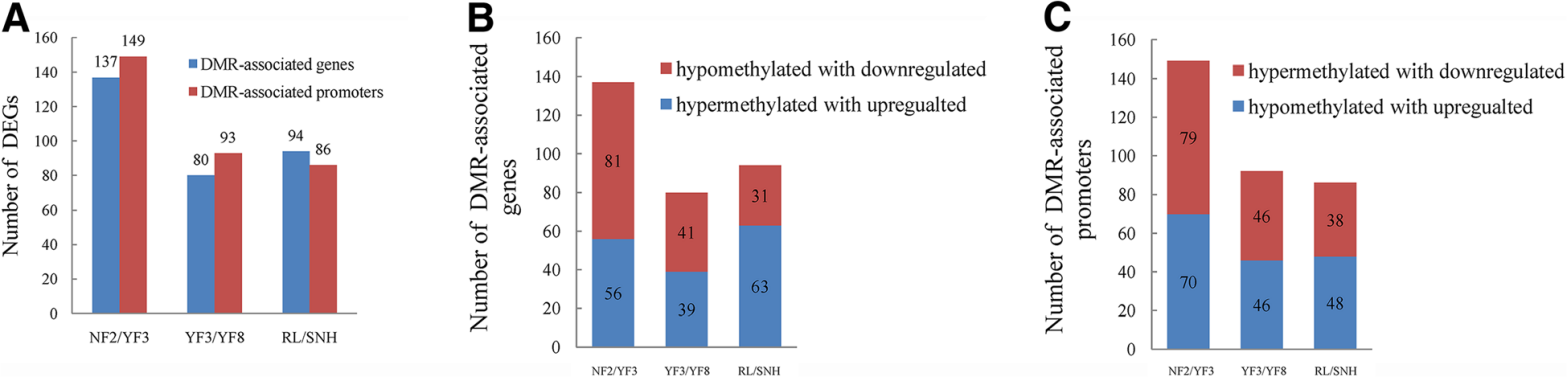
Analysis of differentially expressed genes (DEGs) and differentially methylated regions (DMRs). **a** DEGs identified as DMR-associated genes and promoters in NF2/YF3, YF3/YF8, and RL/SNH comparisons. Numbers of downregulated and upregulated DEGs with hypomethylated and hypermethylated genes (**b**) and promoters (**c**)

To study the influence of DNA methylation on gene expression, we assessed the relationship between DMRs and DEGs on a genome-wide scale. About 6.9% (137), 8.4% (80), and 8.0% (94) of the DEGs in NF22/YF3, YF3/YF8 and RL/SNH, respectively, were identified as DMR-associated genes. Approximately 7.5% (149), 9.7% (93), and 7.3% (86) of the DEGs in the three comparisons, respectively, were identified with DMR-associated promoters (Fig. 6a). We subsequently searched for upregulated genes with hypermethylated gene bodies or hypomethylated promoters among the three comparisons. In NF2/YF3, 56 and 70 upregulated genes had a hypermethylated gene body and a hypomethylated promoter, respectively (39 and 36, respectively, in YF3/YF8; and 63 and 48, respectively, in RL/SNH) (Fig. 6b, c). Downregulated genes with a hypomethylated gene body or hypermethylated promoter were also identified. In NF2/YF3, 81 and 89 downregulated genes had a hypomethylated gene body and a hypermethylated promoter, respectively (41 and 46, respectively, in YF3/YF8; 31 and 38, respectively, in RL/SNH) (Fig. 6b, c).

**Fig. 7:**
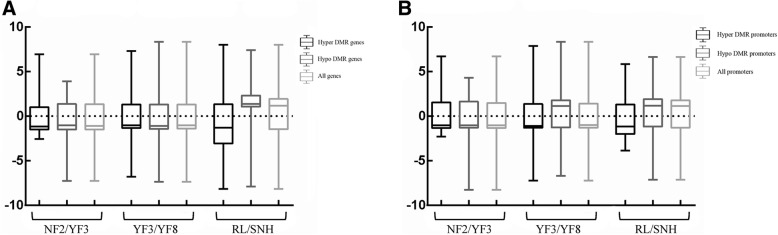
Relationship between differential methylation and gene expression. (**a**) A box-plot of the differential expression of all genes and genes associated with DMRs is displayed. (**b**) A box-plot of the differential expression of all genes and promoters associated with DMRs is displayed. Boxes represent the quartiles; whiskers indicate the date from the min to max

The gene expression patterns and methylation levels were also calculated (Fig. 7). In the three comparisons, the expression of most genes was negatively correlated with gene body methylation (Fig. 7a) and promoter methylation (Fig. 7b). For example, most hypo-DMR-genes in NF2/YF3 lead to downregulate the expression (P<0.05). Most hyper-DMR-promoters also in NF/YF3 lead to downregulate the expression (P<0.05). These results suggested that gene expression changes were not always correlated with the alterations DNA methylation.

### DMR-associated DEGs involved in the anthocyanin pathway

We identified the DMR-associated DEGs involved in anthocyanin synthesis. In NF2/YF3, we detected an upregulated *ANS* (MD06G1071600) with a hypomethylated promoter as well as two downregulated genes, *ABC* (MD13G1109600) and *GST* (MD16G1233100), with hypermethylated promoters. In YF3/YF8, *F3H* (MD05G1074200) was upregulated and had a hypermethylated promoter, whereas *MATE* (MD07G1010700) was downregulated and had a hypomethylated gene body. In RL/SNH, *ANS* (MD06G1071600) was upregulated and had a hypermethylated promoter. These results indicated that the changes to structural genes were influenced by the methylation level, but they may also be regulated by other factors.

Among the non-structural genes, *MYB* genes were found in the three comparisons. Specifically, *MYB114* (MD17G1261100) was downregulated and had a hypomethylated gene body in NF2/YF3, but it was upregulated and had a hypermethylated gene body in YF3/YF8 and RL/SNH. Moreover, *MYB114* (MD17G1261100) was upregulated in YF3/YF8 and RL/SNH, suggesting that it may play a role in regulating anthocyanin biosynthesis in fully red apples. These results suggested that the structural and regulatory genes may be affected by DNA methylation in apple color mutants.

### Validation of DNA methylation and gene expression data

To validate the methylation pattern results, we selected several candidate DMRs for confirmation by bisulfite-PCR and sequencing analyses. Based on our BS-Seq results, we picked at least four DMRs containing genes involved in anthocyanin biosynthesis. These regions were subjected to bisulfite-PCR followed by sequencing for all five apple cultivars. The results of bisulfite-PCR results were consistent with those of the methylome analysis (Additional file 1: Figure S8), suggesting the sequencing results were reliable.

We also validated transcript levels of at least 13 DEGs involved in the anthocyanin pathway in five apple cultivars using real-time PCR analysis. The results indicated that RT-qPCR analyses were consistent with the RNA-Seq data, with similar trends observed for the upregulated and downregulated genes. (Additional file 1: Figure S9).

## Discussion

Apple is one of the most widely cultivated and economically important fruit trees in temperate regions and is very popular with consumers. Fruit colour, which is determined by anthocyanin content, is an important quality in apple. There is increasing evidence that DNA methylation plays an essential role in the regulation of anthocyanin biosynthesis in fruit sport trees [1–3, 23, 24]. Therefore, it is important to explore the DNA methylomes of apple sport cultivars with different red phenotypes to understand the epigenetic regulation of anthocyanin biosynthesis and identify specific markers that contribute to the acquisition of this agronomic trait.

We investigated the dynamics of DNA methylation at a single base resolution by WGBS of five apple cultivars (NF2, YF3, YF8, RL and SNH). Globally, the genome showed 44.3–60.1%, 31.5–44.8%, and 11.8–18.1% methylation levels in the CG, CHG, and CHH contexts, respectively (Additional file 2: Table S6). Previous methylome studies on apple have shown that CG methylation is more common than CHG and CHH methylation. In the five apple cultivars, CG methylation was also the most common type, followed by CHG and CHH methylation. Among the five cultivars, NF2 and YF3 had the highest and lowest methylation levels, respectively. These results suggested that the methylation level is relatively stable in the same species. Previous studies showed that the DNA methylation level is positively related to genome size [26, 27]. Compared with other species (*Arabidopsis*; *Beta vulgaris*; *Eutrema salsugineum*; and *Vitis vinifera*), apple has moderate methylation levels [27]. Our results also demonstrated that the methylation levels in the three contexts were positively correlated with the transposable element density and negatively correlated with gene number, which is consistent with the previously reported results for ‘Qinguan’ and ‘Honeycrisp’ apples [28]. These results suggested that the maintenance of genome stability is a primary function of DNA methylation.

As an epigenetic marker, DNA methylation also helps to repress gene expression. However, the relationship between DNA methylation and gene expression is more complex than initially thought when genome-wide methylome analyses were first conducted. For example, in rice, the methylation of the promoter inhibits gene expression only at highly methylated gene loci and the methylation of the gene body is usually positively correlated with gene expression [29]. Similar results were reported in a study on the *Arabidopsis* methylome [30]. In our study, the expression of most genes was negatively correlated with gene body methylation (Fig. 7a) and with promoter methylation (Fig. 7b). However, the opposite pattern was observed for some DMR-associated DEGs. Indeed, the expression levels of many genes were not correlated with the corresponding methylation level. Similar findings were reported for the ‘Qinguan’ and ‘Honeycrisp’ [28]. Although some downregulated and upregulated genes were associated with hyper and hypo-DMRs, many DEGs did not show significantly different methylation levels. We speculate that the impact of DNA methylation on gene expression may be mediated by both direct and indirect mechanisms. For example, methyl CpG binding domain proteins can recognise mCs regions in promoters and then recruit other transcriptional regulatory proteins to regulate gene expression [31, 32]. Additionally, the mCs may also prevent TFs from binding to the promoter to inhibit gene expression [3, 33]. Meanwhile, the methylation of promoters represses transcription by promoting repressive histone modifications, such as H3K9me2, or inhibiting permissive histone modifications, including histone acetylation [34, 35]. Overall, the gene expression alterations were not always correlated with the corresponding DNA methylation changes. Future studies should aim to clarify how the methylation and demethylation of the promoter and gene body affect transcription in different apple lines.

Anthocyanin biosynthesis in apple is affected by light, temperature, and hormones. In addition to environmental factors, DNA methylation also plays an important role in regulating the anthocyanin pathway. Previous studies reported that the methylation level of the *MYB* promoter is a key factor controlling anthocyanin synthesis in fruit sports. For example, the decreased methylation level of the *MdMYB1* promoter in red ‘Ralls’ was proposed to explain its red colour [23]. Similarly, the methylation level of the *MdMYB10* promoter was proposed to be the epigenetic factor causing the fruit colour variation between ‘Blondee’, which has anthocyanin-deficient yellow skin, and ‘Kidd’s D-8’ [2]. Another study, paper bagging alteres the DNA methylation level and histone modifications at the *MdMYB1* locus and upregulates *MdMYB1* expression, causing differential anthocyanin pigmentation [36]. In this study, we performed WGBS and transcriptome analyses to investigate the mechanism of fruit colour mutations in apple. Our data highlight the differences in DNA methylation among different apple sports. The results show that the DNA methylation levels of both gene body regions and promoter regions may affect anthocyanin biosynthesis via their effects on gene expression. Our analyses also highlighted the following three DMR-associated upregulated DEGs related to the anthocyanin pathway: structural genes *ANS* (MD06G1071600) in NF2/YF3 and RL/SNH and *F3H* (MD05G1074200) in YF3/YF8, as well the TF gene *MYB114* in YF3/YF8 and RL/SNH. While *ANS* in NF2/YF3 was hypomethylated in the promoter region, *F3H* in YF3/YF8 and *ANS* in RL/SNH were hypermethylated in the promoter region. They were both upexpressed in the darker red apple fruits with the different methylation levels. This may have been because that the structural genes, as the downstream of anthocyanin pathway, is primarily regulated by the TFs rather than methylation themselves; or the regulation by methylation themselves was weaker than that by transcriptional control. Additionally, changes in methylation together with transcriptional regulatory activities may act synergistically to enhance the expression of structural genes. Moreover, the expression of a MYB TF gene, *MYB114*, was also found be upregulated both in YF3/YF8 and RL/SNH. The R2R3-MYB transcription factor AtMYB114 combines with bHLH and WD40 to form the MBW complex, which activates structural genes in anthocyanin biosynthesis in *Arabidopsis* [37]. In addition to MYB1, MYB114 may be another regulator of anthocyanin biosynthesis during the apple coloration. The expressions of MYB114 were also upregulated in redder apple fruits. Overall, these results suggest that the deep red colour of apple sport mutants is due to upregulation of structural genes in the anthocyanin pathway. Furthermore, the *MYB114* expression level was found to be upregulated in two blushed apples that indicated that the expression of MYB114 is likely to the one reason from the red-striped to fully red pattern in apple fruits.

## Conclusions

We analysed the methylomes and transcriptomes of deep-red apple mutants and their red-skinned parents, and found DMR-associated DEGs in the anthocyanin pathway in three comparisons (NF2/YF3, YF3/YF8, and RL/SNH). The DEGs included *ANS* (MD06G1071600) in NF2/YF3 and RL/SNH and *F3H* (MD05G1074200) in YF3/YF8. The promoter region was hypomethylated *ANS* in NF2/YF3, whereas *F3H* in YF3/YF8 and *ANS* in RL/SNH had hypermethylated promoter regions. In this study, the differential methylation of structural genes promoters affected the anthocyanin pathway in apple sport mutants, but the methylation was not the primary reason for changes in gene expression. Additionally, we observed that the expression of another MYB TF gene*, MYB114* (MD17G1261100), was upregulated in YF8 and SNH, which have fully red fruit skins. The upregulation of *MYB114* may be the reason that the change from the red-striped to the fully red pattern in apple fruits.

## Methods

### Plant materials and growth conditions

The apple varieties Nagafu 2 (NF2), Yanfu 3 (YF3) and Yanfu 8 (YF8) were grown in Yantai, Shandong Province. The apple varieties Ralls (RL) and Shannonghong (SNH) were obtained from Wulian, Shandong Province. For each variety, ripened fruits were collected from the five varieties. We collected 15 fruits per cultivar. The peels from these fruits were collected, immediately frozen in liquid nitrogen, and then stored at − 80 °C until use. In order to the relationship between the parent and sport, a genetic fingerprinting was performed using 16 simple sequence repeat (SSR) markers (Additional file 2: Table S1).

### Measurement of anthocyanin content

Anthocyanins were extracted with the following two buffers: KCl buffer (0.025 M), pH = 1.0 and NaAc buffer (0.4 M), pH = 4.5. To extract total anthocyanins, 0.5 g finely ground fruit peel was added to 5 ml 1% HCl in methanol (*v*/v) and the mixture was kept at 4 °C in darkness for 24 h. A 1-mL aliquot of this mixture was transferred to a 10 mL centrifuge tube, after which 4 mL KCl buffer was added. A second 1-mL aliquot of the mixture was transferred to a 10-mL centrifuge tube and then 4 mL NaAc buffer was added. This procedure was repeated three times for each sample. Each of the solutions was mixed and kept for 15 min at 4 °C in darkness. The absorbance of the two solutions was measured using a spectrophotometer (UV-1600, Shimadzu, Kyoto, Japan) at 510 and 700 nm, respectively. The anthocyanin content was calculated as following previously published formula [38]:

Anthocyanin content (mg/g FW) = ΔA × 5 × 0.005 × 1000 × 449.2/(26,900 × 0.5); ΔA = (A510 − A700) (pH = 1.0) − (A510 − A700) (pH = 4.5).

### DNA extraction and BS-Seq library construction

Total genomic DNA (gDNA) was extracted from apple peels using a DNeasy Plant Mini Kit (Qiagen, Valencia, CA, USA) according to the manufacturer’s instructions. In total, 4 μg gDNA was cut into 100–500-bp fragments using Bioruptor (Diagenode, Belgium). After end repair, adenylation, and adapter ligation (to protect from bisulfite conversion), the DNA fragments were treated with bisulfite using the ZYMO EZ DNA Methylation-Gold kit (ZYMO Research, Orange County, CA, USA). Target fragments were excised from a 2% TAE agarose gel. The products were purified using a QIAquick Gel Extraction kit (Qiagen) and then amplified by PCR. Finally, the BS-Seq libraries were sequenced on the Illumina HiSeq 4000 platform (BGI-Shenzhen, Shenzhen, China). Each library generated an average of 25 Gb clean bases after filtering to remove low-quality reads, N reads, and adapter sequences.

### BS-Seq data analysis

The raw data were filtered by removing adapter sequences, contamination, and low-quality reads. We used BSMAP [39] to map the sequencing reads back to the apple reference genome (https://iris.angers.inra.fr/gddh13/index.html). The methylation level (%) was computed by dividing the number of reads of each methylated cytosine (mC) by the total reads for that cytosine, as follows:

R_m_ (%) = Nm_all_/(Nm_all_ + Nnm_all_) × 100

where Nm and Nnm represent the read number of mC and nonmethylated-C, respectively. Differentially methylated regions (DMRs) contained at least five CG (CHH or CHG) sites with a 2-fold change (Fisher’s Exact Test, *p* ≤ 0.05) in methylation levels. The DMRs were identified using the sliding-window approach by comparing the parent and mutant methylomes [40]. We used CIRCOS to compare the methylation levels of DMRs among different samples. The degree of difference of a methyl-cytosine (mCG, mCHG, and mCHH) was also calculated using the following formula:

degree of difference = log_2_Rm1/log_2_Rm2

where Rm1 and Rm2 represent the methylation level of mC for sample 1 and sample 2, respectively [the value of Rm1 (or Rm2) was adjusted to 0.001 if it was 0]. After the DMRs were identified, the genes and promoters located in the DMRs were characterised. The DMR-associated genes and promoters were analysed using Gene Ontology (GO) and Kyoto Encyclopedia of Genes and Genomes (KEGG) enrichment tools. The GO analysis was performed with the GO::TermFinder software [41]. The calculated *p*-value was subjected to Bonferroni correction. The GO terms with a *p-*value ≤0.05 were defined as significantly enriched with DMR-related genes or promoters. A pathway enrichment analysis of DMR-related genes was conducted using KEGG [42]. Pathways with a *p*-value ≤0.05 were regarded as significantly enriched.

### RNA extraction and transcriptome sequencing

Total RNAs were isolated using an RNAprep pure Plant Kit (Tiangen, Beijing, China) from the same apple peel samples used in the methylome analysis. Three biological replicates for each sample were used for the transcriptome analysis. The RNA concentration and quality were tested using NanoDrop (Thermo Fisher Scientific, Waltham, MA, USA) and Agilent 2100 instruments (Agilent Technologies, Santa Clara, CA, USA). We used 5 μg RNA per sample for library construction. The libraries were sequenced by the BGISEQ-500 platform (BGI, Shenzhen, China). After removing the adapter and low-quality reads, clean reads were aligned to the apple genome (https://iris.angers.inra.fr/gddh13/index.html) using the HISAT and Bowtie2 tools [43, 44]. For gene expression analyses, FPKM values were calculated using the RESM software [45]. Differentially expressed genes (DEGs) were identified with the NOISeq software, in accordance with the following criteria: fold change ≥2 and diverge probability ≥0.8 [46]. The DEGs were also analysed using GO and KEGG tools.

### RNA isolation and RT-PCR analysis for gene expression

Total RNAs were isolated using an RNAprep pure Plant Kit (Tiangen), following the manufacturer’s instructions. First-strand cDNA synthesis was conducted using a TransScript II One-Step gDNA Removal kit and a cDNA Synthesis SuperMix Kit (TransGen, Beijing, China). The primers for RT-PCR were designed using Beacon Designer 7 software and synthesised by Sangon Biotech Co. (Shanghai, China). The primers are listed in Additional file 2: Table S7. The reactions for RT-PCR were performed using cDNAs as templates with a qPCR SuperMix Kit (TransGen). Three biological and three technical replicates for each reaction were analysed on a CFX96 instrument (BioRad, Hercules, CA, USA). The thermal cycling conditions were as follows: 94 °C for 30 s followed by 40 cycles of 94 °C for 5 s, 58 °C for 15 s, and 72 °C for 10 s. A melting curve was performed for each sample at the end of each run. Relative expression was calculated using the cycle threshold (Ct) 2^-ΔΔCt^ method [47].

### Bisulfite sequencing PCR (BSP) analysis

A BSP analysis was carried out to validate the BS-Seq results [1]. We extracted 750 ng *g*DNA from fruit skin samples of ‘Nagafu 2’, ‘Yanfu 3’, ‘Yanfu 8’, ‘Ralls’ and ‘Shannonghong’. The gDNA was treated with an EZ DNA Methylation-Gold Kit (Zymo Research). Degenerate primers were used to amplify the target fragments from the treated *g*DNA using the Ex Taq® Hot Start Version (TaKaRa, Otsu, Japan). All fragments were ligated into the pEasy blunt zero vector (TransGen), and then sequenced by Sangon Biotech Co. (Shanghai, China). For each fragment with three independent PCR reactions generated 12 independent clones for sequencing. The results were analysed using the online software Kismeth [48]. The primers used for BSP are listed in Additional file 2: Table S7.

### Statistical analyses

The qRT-PCR and BS-PCR results shown in figures are the mean of three replicated treatments with three biological repetitions. Significant differences among samples were statistically evaluated by standard deviation and analysis of variance (ANOVA). Statistical analyses were performed with the SPSS 12.0 software (IBM Corp., Armonk, NY, USA).

## Additional files

Additional file 1: **Figure S1.** Evaluation of the relationships among five apple cultivars. **Figure S2.** Summary of sequencing and mapping. **Figure S3.** Global methylome maps of apple cultivars. **Figure S4.** Distribution of mCs on the chromosomes of five apple cultivars. **Figure S5.** KEGG analysis of DMRs in three comparisons. **Figure S6.** Differential gene expression in three comparisons. **Figure S7.** Gene Ontology (GO) and KEGG pathway analyses of the DEGs among three comparisons. **Figure S8.** Validation of DNA methylation results in three comparisons. **Figure S9.** Validation of the expression level of candidate genes related to the anthocyanin biosynthetic pathway in five cultivars. (DOCX 255518 kb)

Additional file 2: **Table S1.** Details of the bisulfite sequencing libraries generated in the study. **Table S2.** Effective coverage of intergenic region. **Table S3.** Distribution of DMRs on chromosomes. **Table S4.** Summary of the sequencing data generated for RNA-Seq and mapping on the apple genome. **Table S5.** List of differentially expressed genes involved in the anthocyanin pathway in the three comparisons. **Table S6.** Average methylation level of five apple cultivars. **Table S7.** Primers for qRT-PCR and BS-PCR. (DOCX 34 kb)

## Abbreviations

ABC: ATP-binding cassette
ANS: Leucoanthocyanidin dioxygenase
CHI: Chalcone isomerise
CHS: Chalcone synthase
CMT2, 3: CHROMOMETHYLASE2, 3
DDM1: DECREASE IN DNA METHYLATION 1
DEG: Differentially expressed gene
DFR: Dihydroflavonol-4-reductase
DME: DEMETER
DML2: DEMETER-LIKE 2
DMR: Differentially methylated region
DRM2: DOMAINS REARRANGED METHYLTRANSFERASE 2
F3H: Flavanone-3-hydroxylase
GST: Glutathione S-transferase
MATE: Multidrug and toxic compound extrusion
mCs: Methylcytosines
MET1: METHYLTRANSFERASE 1
NF2: Nagafu 2
ROS1: REPRESSOR OF SILENCING 1
TF: Transcription factors
UFGT: UDP-glucose:flavonoid 3-O-glucosyltransferase
YF3: Yanfu 3
YF8: Yanfu 8

## References

[1] Telias A, Lin-Wang K, Stevenson DE, Cooney JM, Hellens RP, Allan AC, Hoover EE, Bradeen JM. Apple skin patterning is associated with differential expression of MYB10. BMC Plant Biol. 2011;11:93. 10.1186/1471-2229-11-93 PMC3127826 21599973

[2] El-Sharkawy I, Liang D, Xu K (2015). Transcriptome analysis of an apple (Malus x domestica) yellow fruit somatic mutation identifies a gene network module highly associated with anthocyanin and epigenetic regulation. J Exp Bot.

[3] Wang Z, Meng D, Wang A, Li T, Jiang S, Cong P, Li T (2013). The methylation of the PcMYB10 promoter is associated with green-skinned sport in max red Bartlett pear. Plant Physiol.

[4] Hichri I, Deluc LG, Barrieu F, Bogs J, Mahjoub A, Regad F, Gallois B, Granier T, Trossatmagnin C (2011). A single amino acid change within the R2 domain of the VvMYB5b transcription factor modulates affinity for protein partners and target promoters selectivity. BMC Plant Biol.

[5] Xi X, Zha Q, Jiang A, Tian Y (2016). Impact of cluster thinning on transcriptional regulation of anthocyanin biosynthesis-related genes in ‘summer black’ grapes. Plant Physiol Biochem.

[6] Winkelshirley B (2001). Flavonoid biosynthesis. A colorful model for genetics, biochemistry, cell biology, and biotechnology. Plant Physiol.

[7] Takos AM, Jaffe FW, Jacob SR, Bogs J, Robinson SP, Walker AR (2006). Light-induced expression of a MYB gene regulates anthocyanin biosynthesis in red apples. Plant Physiol.

[8] Ban Y, Honda C, Hatsuyama Y, Igarashi M, Bessho H, Moriguchi T (2007). Isolation and functional analysis of a MYB transcription factor gene that is a key regulator for the development of red coloration in apple skin. Plant Cell Physiol.

[9] Xie XB, Li S, Zhang RF, Zhao J, Chen YC, Zhao Q, Yao YX, You CX, Zhang XS, Hao YJ (2012). The bHLH transcription factor MdbHLH3 promotes anthocyanin accumulation and fruit colouration in response to low temperature in apples. Plant Cell Environ.

[10] An XH, Tian Y, Chen KQ, Wang XF, Hao YJ (2012). The apple WD40 protein MdTTG1 interacts with bHLH but not MYB proteins to regulate anthocyanin accumulation. J Plant Physiol.

[11] Lin-Wang K, Micheletti D, Palmer J, Volz R, Lozano L, Espley R, Hellens RP, Chagne D, Rowan DD, Troggio M (2011). High temperature reduces apple fruit colour via modulation of the anthocyanin regulatory complex. Plant Cell Environ.

[12] An XH, Tian Y, Chen KQ, Liu XJ, Liu DD, Xie XB, Cheng CG, Cong PH, Hao YJ (2015). MdMYB9 and MdMYB11 are involved in the regulation of the JA-induced biosynthesis of anthocyanin and proanthocyanidin in apples. Plant Cell Physiol..

[13] Liu XJ, An XH, Liu X, Hu DG, Wang XF, You CX, Hao YJ (2017). MdSnRK1.1 interacts with MdJAZ18 to regulate sucrose-induced anthocyanin and proanthocyanidin accumulation in apple. J Exp Bot.

[14] Cedar H, Bergman Y (2012). Programming of DNA methylation patterns. Annu Rev Biochem.

[15] Law JA, Jacobsen SE (2010). Establishing, maintaining and modifying DNA methylation patterns in plants and animals. Nat Rev Genet.

[16] Cao X, Jacobsen SE (2002). Locus-specific control of asymmetric and CpNpG methylation by the DRM and CMT3 methyltransferase genes. PNAS.

[17] Stroud H, Do T, Du J, Zhong X, Feng S, Johnson L, Patel DJ, Jacobsen SE (2013). Non-CG methylation patterns shape the epigenetic landscape in Arabidopsis. Nat Struct Mol Biol.

[18] Jeddeloh JA, Stokes TL, Richards EJ (1999). Maintenance of genomic methylation requires a SWI2/SNF2-like protein. Nat Genet.

[19] Choi Y, Gehring M, Johnson L, Hannon M, Harada JJ, Goldberg RB, Jacobsen SE, Fischer RL (2002). DEMETER, a DNA glycosylase domain protein, is required for endosperm gene imprinting and seed viability in Arabidopsis. Cell.

[20] Zhizhong G, Teresa MR, Ariza RR, Teresa RA, Lisa D, Jian Kang Z (2002). ROS1, a repressor of transcriptional gene silencing in Arabidopsis, encodes a DNA glycosylase/lyase. Cell.

[21] Penterman J, Zilberman D, Huh JH, Ballinger T, Henikoff S, Fischer RL (2007). DNA demethylation in the Arabidopsis genome. PNAS.

[22] Chen X, Guo W, Xu J, Cong P, Wang L, Liu C (2015). Genetic improvement and promotion of fruit quality of main fruit trees. Sci Agric Sin.

[23] Xu Y, Feng S, Jiao Q, Liu C, Zhang W, Chen W, Chen X (2011). Comparison of MdMYB1 sequences and expression of anthocyanin biosynthetic and regulatory genes between Malus domestica Borkh. Cultivar ‘Ralls’ and its blushed sport. Euphytica.

[24] Qian M, Sun Y, Allan AC, Teng Y, Zhang D (2014). The red sport of ‘Zaosu’ pear and its red-striped pigmentation pattern are associated with demethylation of the PyMYB10 promoter. Phytochemistry.

[25] Daccord N, Celton J-M, Linsmith G, Becker C, Choisne N, Schijlen E, van de Geest H, Bianco L, Micheletti D, Velasco R (2017). High-quality de novo assembly of the apple genome and methylome dynamics of early fruit development. Nat Genet.

[26] Ausin I, Feng S, Yu C, Liu W, Kuo HY, Jacobsen EL, Zhai J, Gallego-Bartolome J, Wang L, Egertsdotter U (2016). DNA methylome of the 20-gigabase Norway spruce genome. Proc Natl Acad Sci.

[27] Niederhuth CE, Bewick AJ, Ji L, Alabady MS, Kim KD, Li Q, Rohr NA, Rambani A, Burke JM, Udall JA, et al. Widespread natural variation of DNA methylation within angiosperms. Genome Biol. 2016;17:194. 10.1186/s13059-016-1059-0 PMC5037628 27671052

[28] Xu J, Zhou S, Gong X, Song Y, van Nocker S, Ma F, Guan Q (2018). Single-base methylome analysis reveals dynamic epigenomic differences associated with water deficit in apple. Plant Biotechnol J.

[29] Li X, Zhu J, Hu F, Ge S, Ye M, Xiang H, Zhang G, Zheng X, Zhang H, Zhang S (2012). Single-base resolution maps of cultivated and wild rice methylomes and regulatory roles of DNA methylation in plant gene expression. BMC Genomics.

[30] Meng D, Dubin MJ, Zhang P, Osborne EJ, Stegle O, Clark RM, Nordborg M. Limited contribution of DNA methylation variation to expression regulation in Arabidopsis thaliana. PLoS Genet. 2016;112(7):e1006141. 10.1371/journal.pgen.1006141 PMC4939946 27398721

[31] Clouaire T, Stancheva I (2008). Methyl-CpG binding proteins: specialized transcriptional repressors or structural components of chromatin?. Cell Mol Life Sci.

[32] Li Y, Deng H, Miao M, Li H, Huang S, Wang S, Liu Y (2016). Tomato MBD5, a methyl CpG binding domain protein, physically interacting with UV-damaged DNA binding protein-1, functions in multiple processes. New Phytol.

[33] Bird A (2002). DNA methylation patterns and epigenetic memory. Genes Dev.

[34] Domcke S, Bardet AF, Adrian Ginno P, Hartl D, Burger L, Schübeler D (2015). Competition between DNA methylation and transcription factors determines binding of NRF1. Nature.

[35] Zhu H, Wang G, Qian J (2016). Transcription factors as readers and effectors of DNA methylation. Nat Rev Genet.

[36] Bai S, Tuan PA, Saito T, Honda C, Hatsuyama Y, Ito A, Moriguchi T (2016). Epigenetic regulation of MdMYB1 is associated with paper bagging-induced red pigmentation of apples. Planta.

[37] Gonzalez A, Zhao M, Leavitt JM, Lloyd AM (2008). Regulation of the anthocyanin biosynthetic pathway by the TTG1/bHLH/Myb transcriptional complex in Arabidopsis seedlings. Plant J.

[38] Jin W, Wang H, Li M, Wang J, Yang Y, Zhang X, Yan G, Zhang H, Liu J, Zhang K (2016). The R2R3 MYB transcription factor PavMYB10.1 involves in anthocyanin biosynthesis and determines fruit skin colour in sweet cherry (Prunus avium L.). Plant Biotechnol J.

[39] Xi Y, Li W (2009). BSMAP: whole genome bisulfite sequence MAPping program. BMC Bioinformatics..

[40] Heyn H, Li N, Ferreira HJ, Moran S, Pisano DG, Gomez A, Diez J, Sanchezmut JV, Setien F, Carmona FJ (2012). Distinct DNA methylomes of newborns and centenarians. PNAS.

[41] Boyle EI, Weng S, Gollub J, Jin H, Botstein D, Cherry JM, Sherlock G (2004). GO: :TermFinder---open source software for accessing gene ontology information and finding significantly enriched gene ontology terms associated with a list of genes. Bioinformatics.

[42] Kanehisa M, Araki M, Goto S, Hattori M, Hirakawa M, Itoh M, Katayama T, Kawashima S, Okuda S, Tokimatsu T (2007). KEGG for linking genomes to life and the environment. Nucleic Acids Res.

[43] Kim D, Langmead B, Salzberg SL (2015). HISAT: a fast spliced aligner with low memory requirements. Nat Methods.

[44] Langmead B, Trapnell C, Pop M, Salzberg SL. Ultrafast and memory-efficient alignment of short DNA sequences to the human genome. Genome Biol. 2009;10(3). 10.1186/gb-2009-10-3-r25 PMC2690996 19261174

[45] Li B, Dewey CN (2011). RSEM: accurate transcript quantification from RNA-Seq data with or without a reference genome. BMC Bioinformatics.

[46] Tarazona S, Garciaalcalde F, Dopazo J, Ferrer A, Conesa A (2011). Differential expression in RNA-seq: a matter of depth. Genome Res.

[47] Livak KJ, Schmittgen TD (2001). Analysis of relative gene expression data using real-time quantitative PCR and the 2(−Delta Delta C(T)) method. Methods.

[48] Gruntman E, Qi Y, Slotkin RK, Roeder T, Martienssen RA, Sachidanandam R (2008). Kismeth:analyze of plant methylation states through bisulfitesequencing. BMC Bioinformatics..

