# Supplementary material for: Methylome and transcriptome analyses of apple fruit somatic mutations reveal the difference of red phenotype
Source: BMC Genomics. 2019 Feb 7;20:117. doi: 10.1186/s12864-019-5499-2 (PMC6367808; doi:10.1186/s12864-019-5499-2)
Supplement: Supplementary file 2 — Table S1. Details of the bisulfite sequencing libraries generated in the study. Table S2. Effective coverage of intergenic region. Table S3. Distribution of DMRs on chromosomes. Table S4. Summary of the sequencing data generated for RNA-Seq and mapping on the apple genome. Table S5. List of differentially expressed genes involved in the anthocyanin pathway in the three comparisons. Table S6. Average methylation level of five apple cultivars. Table S7. Primers for qRT-PCR and BS-PCR. (DOCX 34 kb) [file 12864_2019_5499_MOESM2_ESM.docx]

**Table S1.** Details of the bisulfite sequencing libraries generated in the study.

| Sample | Clean Reads number | Mapped Reads number | Mapping Rate  (%) | Uniquely Mapped Reads | Uniquely Mapping Rate (%) | Bisulfite Conversion Rate (%) |
| --- | --- | --- | --- | --- | --- | --- |
| NF2 | 250000004 | 162443704 | 64.98 | 142453711 | 56.98 | 99.57 |
| YF3 | 250000004 | 197099177 | 78.84 | 171717590 | 68.69 | 99.58 |
| YF8 | 250000004 | 182789070 | 73.12 | 159594606 | 63.84 | 99.58 |
| RL | 250000004 | 216465923 | 86.59 | 187657786 | 75.06 | 99.4 |
| SNH | 250000002 | 217667799 | 87.07 | 187344917 | 74.94 | 99.43 |

NF2, YF3, YF8, RL and SNH represent Nagafu 2, Yanfu 3, Yanfu 8, Ralls and Shannonghong apple cultivars, respectively. Bisulfite Conversion Rate = 1 - methylation rate of Lambda DNA

**Table S2.** Effective coverage of intergenic region.

| Location | C | | | CG | | | | CHG | | | | CHH | | | |
| --- | --- | --- | --- | --- | --- | --- | --- | --- | --- | --- | --- | --- | --- | --- | --- |
|  | NF2 | YF3 | YF8 | | NF2 | YF3 | YF8 | | NF2 | YF3 | YF8 | | NF2 | YF3 | YF8 |
| 3-UTR | 96.2 | 95.9 | 96.5 | | 96.2 | 95.8 | 96.4 | | 96.5 | 96.1 | 96.7 | | 96.2 | 95.8 | 96.5 |
| 5-UTR | 93.4 | 93.2 | 94.3 | | 94.9 | 94.4 | 95.3 | | 95.0 | 94.6 | 95.5 | | 92.7 | 92.6 | 93.7 |
| CDS | 96.4 | 96.0 | 96.6 | | 96.5 | 96.0 | 96.6 | | 96.8 | 96.4 | 97.0 | | 96.3 | 95.9 | 96.5 |
| CpG Island | 84.0 | 79.9 | 83.4 | | 84.7 | 80.8 | 84.0 | | 84.4 | 80.3 | 83.7 | | 83.5 | 79.4 | 83.1 |
| intron | 90.2 | 88.8 | 90.4 | | 87.4 | 85.5 | 87.6 | | 90.5 | 89.1 | 90.7 | | 90.4 | 89.1 | 90.7 |
| mRNA | 93.4 | 92.5 | 93.6 | | 93.1 | 92.1 | 93.2 | | 94.2 | 93.3 | 94.3 | | 93.2 | 92.3 | 93.6 |
| repeat | 86.7 | 82.8 | 86.9 | | 86.1 | 82.0 | 86.0 | | 85.9 | 81.8 | 85.9 | | 86.9 | 83.1 | 87.2 |

| Location | C | | CG | | CHG | | CHH | |
| --- | --- | --- | --- | --- | --- | --- | --- | --- |
|  | RL | SNH | RL | SNH | RL | SNH | RL | SNH |
| 3-UTR | 96.0 | 96.1 | 95.9 | 96.1 | 96.2 | 96.3 | 96.0 | 96.0 |
| 5-UTR | 92.5 | 91.9 | 94.1 | 91.9 | 94.3 | 94.1 | 91.7 | 91.0 |
| CDS | 96.1 | 96.1 | 96.1 | 96.1 | 96.6 | 96.6 | 96.0 | 96.0 |
| CpG Island | 80.6 | 81.8 | 81.7 | 81.8 | 81.3 | 82.6 | 79.9 | 81.1 |
| intron | 89.7 | 89.8 | 86.6 | 89.8 | 90.0 | 90.3 | 90.0 | 90.1 |
| mRNA | 92.9 | 93.0 | 92.5 | 93.0 | 93.7 | 93.9 | 92.8 | 92.8 |
| repeat | 85.1 | 85.5 | 84.3 | 85.5 | 84.0 | 84.6 | 85.4 | 85.8 |

**Table S3.** Distribution of DMRs on chromosomes.

| Chromosome  (NF2/YF3) | Numbers of DMR | Length of DMR region | Numbers of DMR | Length of DMR region | Numbers of DMR | Length of DMR region |
| --- | --- | --- | --- | --- | --- | --- |
|  | CG | | CHG | | CHH | |
| Chr1 | 360 | 119023 | 236 | 60892 | 2 | 465 |
| Chr2 | 465 | 149957 | 299 | 77913 | 0 | 0 |
| Chr3 | 520 | 166419 | 370 | 96740 | 3 | 648 |
| Chr4 | 381 | 120846 | 291 | 72534 | 1 | 249 |
| Chr5 | 601 | 198112 | 401 | 103502 | 6 | 934 |
| Chr6 | 412 | 127455 | 249 | 65581 | 0 | 0 |
| Chr7 | 499 | 159297 | 339 | 88852 | 1 | 222 |
| Chr8 | 430 | 139983 | 269 | 69540 | 8 | 1536 |
| Chr9 | 473 | 153284 | 317 | 83183 | 7 | 1347 |
| Chr10 | 480 | 154644 | 372 | 97777 | 6 | 1272 |
| Chr11 | 588 | 190656 | 352 | 84197 | 2 | 486 |
| Chr12 | 427 | 142326 | 282 | 72425 | 3 | 620 |
| Chr13 | 419 | 138336 | 294 | 76949 | 5 | 935 |
| Chr14 | 406 | 131460 | 261 | 71746 | 4 | 853 |
| Chr15 | 647 | 207808 | 436 | 107687 | 5 | 1101 |
| Chr16 | 411 | 131599 | 277 | 70162 | 8 | 1494 |
| Chr17 | 448 | 144988 | 326 | 81828 | 6 | 867 |
| Total | 7967 | 2576193 | 5371 | 1381508 | 67 | 13029 |

| Chromosome  (YF3/YF8) | Numbers of DMR | Length of DMR region | Numbers of DMR | Length of DMR region | Numbers of DMR | Length of DMR region |
| --- | --- | --- | --- | --- | --- | --- |
|  | CG | | CHG | | CHH | |
| Chr1 | 302 | 98759 | 223 | 56217 | 2 | 429 |
| Chr2 | 431 | 141422 | 275 | 70978 | 6 | 864 |
| Chr3 | 486 | 151327 | 366 | 96540 | 5 | 902 |
| Chr4 | 365 | 119312 | 255 | 69326 | 5 | 1049 |
| Chr5 | 574 | 183054 | 432 | 107692 | 3 | 716 |
| Chr6 | 330 | 105840 | 231 | 59706 | 4 | 867 |
| Chr7 | 454 | 142334 | 374 | 97146 | 2 | 456 |
| Chr8 | 435 | 144976 | 314 | 84623 | 19 | 5532 |
| Chr9 | 411 | 136943 | 323 | 82808 | 3 | 642 |
| Chr10 | 472 | 153701 | 367 | 96780 | 6 | 1260 |
| Chr11 | 572 | 187322 | 335 | 87873 | 1 | 205 |
| Chr12 | 381 | 128036 | 287 | 76461 | 5 | 883 |
| Chr13 | 342 | 112289 | 274 | 74059 | 2 | 468 |
| Chr14 | 409 | 134560 | 295 | 78265 | 5 | 939 |
| Chr15 | 624 | 203337 | 397 | 101136 | 10 | 1987 |
| Chr16 | 406 | 126871 | 281 | 74016 | 3 | 498 |
| Chr17 | 386 | 125504 | 293 | 76107 | 8 | 1576 |
| Total | 7650 | 2484110 | 5640 | 1474234 | 94 | 19991 |

| Chromosome  (RL/SNH) | Numbers of DMR | Length of DMR region | Numbers of DMR | Length of DMR region | Numbers of DMR | Length of DMR region |
| --- | --- | --- | --- | --- | --- | --- |
|  | CG | | CHG | | CHH | |
| Chr1 | 292 | 99197 | 184 | 48905 | 2 | 221 |
| Chr2 | 362 | 128326 | 255 | 68912 | 2 | 232 |
| Chr3 | 446 | 149265 | 264 | 67932 | 2 | 422 |
| Chr4 | 360 | 119408 | 198 | 55005 | 4 | 441 |
| Chr5 | 460 | 152278 | 326 | 86102 | 5 | 868 |
| Chr6 | 328 | 112520 | 204 | 54080 | 3 | 722 |
| Chr7 | 461 | 160645 | 291 | 76451 | 3 | 435 |
| Chr8 | 389 | 127919 | 261 | 72148 | 5 | 1173 |
| Chr9 | 364 | 118446 | 257 | 70670 | 3 | 694 |
| Chr10 | 428 | 145890 | 257 | 73047 | 2 | 426 |
| Chr11 | 455 | 153048 | 304 | 78105 | 2 | 454 |
| Chr12 | 341 | 120190 | 195 | 57965 | 1 | 451 |
| Chr13 | 365 | 119798 | 230 | 59216 | 0 | 0 |
| Chr14 | 338 | 111718 | 237 | 63375 | 3 | 424 |
| Chr15 | 582 | 192696 | 382 | 99299 | 7 | 1094 |
| Chr16 | 306 | 104452 | 209 | 57267 | 4 | 827 |
| Chr17 | 327 | 111958 | 219 | 58143 | 0 | 0 |
| Total | 6604 | 2227754 | 4273 | 1146622 | 48 | 8884 |

**Table S4.** Summary of the sequencing data generated for RNA-Seq and mapping on the apple genome.

| Sample | Raw Reads | Clean Reads | Mapped Reads |
| --- | --- | --- | --- |
| NF2-1 | 24137307 | 23986897 | 17009108 |
| NF2-2 | 24137047 | 23824379 | 16943898 |
| NF2-3 | 24137014 | 24095430 | 17167993 |
| YF3-1 | 24136849 | 24069735 | 17349464 |
| YF3-2 | 24136893 | 24074145 | 17446532 |
| YF3-3 | 24137179 | 24076475 | 17467482 |
| YF8-1 | 24136970 | 24100614 | 17472945 |
| YF8-2 | 24136904 | 24097546 | 17343003 |
| YF8-3 | 24136937 | 23981583 | 17254748 |
| RL-1 | 24137135 | 24103502 | 17019482 |
| RL-2 | 24136871 | 23925168 | 16809823 |
| RL-3 | 24136805 | 24095608 | 16900659 |
| SNH-1 | 24137069 | 24071465 | 16927054 |
| SNH-2 | 24136761 | 24100415 | 17019713 |
| SNH-3 | 24137256 | 24072464 | 16973494 |

NF2, YF3, YF8, RL and SNH represent Nagafu 2, Yanfu 3, Yanfu 8, Ralls and Shannonghong apple cultivars, respectively.

**Table S5.** List of differentially expressed genes involved in the anthocyanin pathway in the three comparisons.

| Function | Term | Annoation | NF2/YF3 | YF3/YF8 | RL/SNH |
| --- | --- | --- | --- | --- | --- |
| synthesis | CHS | chalcone synthase | MD04G1003300 MD13G1285100 | MD04G1003300  MD04G1003400 | MD13G1285100 |
|  | CHI | chalcone--flavonone isomerase |  | MD01G1167300  MD07G1233400 | MD01G1167300 |
|  | DFR | bifunctional dihydroflavonol 4-reductase/flavanone 4-reductase | MD15G1246200 |  |  |
|  | ANS | leucoanthocyanidin dioxygenase | MD03G1001100  MD06G1211400 MD06G1071600 | MD03G1001100  MD06G1211400 | MD03G1001100 MD06G1071600 |
|  | UFGT | anthocyanidin 3-O-glucosyltransferase | MD17G1055500 | MD01G1234400  MD07G1306900  MD17G1055500 | MD01G1234400 |
| transport | GST | glutathione S-transferase | MD04G1139400 MD05G1184200  MD05G1184300  MD06G1012200  MD10G1196300  MD13G1081900  MD16G1233100 MD17G1134300 | MD10G1172100  MD17G1272100 | MD03G1282900  MD05G1184600  MD05G1210700  MD06G1223000  MD10G1172100  MD10G1172300  MD10G1196600  MD14G1232100  MD16G1081100  MD17G1272100 |
|  | MATE | MATE efflux family | MD16G1034100 | MD07G1010700 | MD04G1165300 |
|  | ABC | ABC transporter | MD13G1109600  MD16G1272900  MD17G1049500 | MD00G1161600  MD09G1265000  MD13G1109600 | MD04G1190300  MD09G1204200  MD09G1265000  MD09G1204300 MD11G1314300  MD13G1109600 |

**Table S6.** Average methylation level of five apple cultivars.

| Varieties | C | CG | CHG | CHH |
| --- | --- | --- | --- | --- |
| NF2 | 26.8 | 60.1 | 44.8 | 18.1 |
| YF3 | 18.4 | 44.3 | 31.5 | 11.8 |
| YF8 | 25.4 | 57.8 | 42.3 | 17.3 |
| RL | 21.3 | 52.3 | 38.2 | 13.5 |
| SNH | 21.5 | 53.0 | 38.9 | 13.4 |

**Table S7.** Primers for qRT-PCR and BS-PCR

|  | Gene | Annotation | | | | Forward | | Reverse |
| --- | --- | --- | --- | --- | --- | --- | --- | --- |
| qRT-PCR | MD04G1003300 | | | CHS | GGACTGGAACTCACTCTT | | TACCGTAATCCGACAACA | |
|  | MD13G1285100 | | | CHS | GGAACTCACTCTTCTGGAT | | GCCGTAATCTGACAACAC | |
|  | MD01G1167300 | | | CHI | TTCTGGTGAACTTGAGAGT | | GGAATGTGATGACGGAATC | |
|  | MD03G1001100 | | | ANS | TTGAGCAGAAGGAGAAGTAT | | CAATGTAATCAGCAGGTGTT | |
|  | MD01G1234400 | | | UFGT | CCCATACCAAACGACCTA | | TATTGCCATCTGCTCCTT | |
|  | MD17G1055500 | | | UFGT | GGTCAGTGCTCTATGTCA | | CTCCAATAATTCCTCTCCAAG | |
|  | MD10G1172100 | | | GST | CCTCGCTTGTGTAATTGG | | CCCTCCTCTGCTTGTAAA | |
|  | MD17G1272100 | | | GST | AGTTGTAGAAGATGGTGACT | | CAGGTCGTTGAAGTTGTG | |
|  | MD05G1184300 | | | GST | CCAGAGTCCACTATCATTCT | | TCCTGCTCTTCACCAATT | |
|  | MD07G1010700 | | | MATE | CACAGCATTCATTGGTCATA | | TATCTTCCTCGGCAACAA | |
|  | MD04G1165300 | | | MATE | TCATTGCTTCTCCGTCTT | | TCCTGCTTCTTCTCATCC | |
|  | MD13G1109600 | | | ABC | GCATCATCATCCTTATGTCTC | | TCTCTGGCTGTGGTAATG | |
|  | MD17G1261100 | | | MYB114 | GCATTGAGATTCATGGAGAA | | TGTGAAGCCTAATCGTAAGA | |
| BS-PCR | MD05G1074200 | | F3H | | GATAATGATTGTTTTTTTTTATTTTYGG | | CATATAAATAAAAAAATA  AAATAAAAAATAAAA | |
|  |  |  |  |  | GTATGGTATAAATATTGGATAATAATGTTA | | AAAATAAAATTATTAATGGTATAAGAAA | |
|  |  |  |  |  | GGATAATATAATTTTGATGATATGGAG | | CTAATTATAATTTTAAAAAATTRCTAATAAAC | |
|  | MD06G1071600 | | ANS | | GTTAATTTTTGGTTGYATGATGA | | TTAATAATATAACRTAATTTTATAATAAATAATTAA | |
|  | MD07G1010700 | | MATE | | GGGTTTGGTGTGTAAGTGAGTTATT | | ATATTCATTTTTRCAACAATATCTTCCT | |
|  |  |  |  |  | TGGTTTTAAAATTATTATGGAAAAG | | CAACAATAACATATAAAAAAATTTTTATATCC | |
|  | MD13G1109600 | | ABC | | GTATAGGATAATTTGATAAGAATTTTTTT | | CCAATTATATTRCTTAACTCAAATCACT | |
|  |  |  |  |  | GAAATTAAGTAATTAATTATGTTGTGAG | | TTCTCTCTCTCTCTCTCTAAAACTATAA | |
|  |  |  |  |  | GTTTTTTAGTTTGTGTTTTATTGTTT | | CAATAAAAAAAATAAATTCAATAACTTTT | |
|  | MD16G1247600 | | bHLH | | AGTATAAATATTATTGGGAAATTAATATGT | | AAAAAACCATAAAAAAAACTACTCC | |
|  |  |  |  |  | TGGATAAGGTTTTTGTAATTAAAGA | | TATTTCAATTTTTATCTTCAAATAATCT | |
|  | MD17G1261100 | | MYB114 | | TGGAGGGATGTAAYGTTAATAAGAG | | AAATCTTATCACAATATTCTTTCTCATTT | |
