# Supplementary material for: Methylome and transcriptome analyses of apple fruit somatic mutations reveal the difference of red phenotype
Source: BMC Genomics. 2019 Feb 7;20:117. doi: 10.1186/s12864-019-5499-2 (PMC6367808; doi:10.1186/s12864-019-5499-2)
Supplement: Supplementary file 1 — Figure S1. Evaluation of the relationships among five apple cultivars. Figure S2. Summary of sequencing and mapping. Figure S3. Global methylome maps of apple cultivars. Figure S4. Distribution of mCs on the chromosomes of five apple cultivars. Figure S5. KEGG analysis of DMRs in three comparisons. Figure S6. Differential gene expression in three comparisons. Figure S7. Gene Ontology (GO) and KEGG pathway analyses of the DEGs among three comparisons. Figure S8. Validation of DNA methylation results in three comparisons. Figure S9. Validation of the expression level of candidate genes related to the anthocyanin biosynthetic pathway in five cultivars. (DOCX 255518 kb) [file 12864_2019_5499_MOESM1_ESM.docx]

**Fig. S1.** Evaluation of the relationships among five apple cultivars. 16 SSR primers were used to evacuate the relationship among five cultivars. The amplicons were visualized by agarose gel (1%) electrophoresis。

**Fig. S2.** Summary of sequencing and mapping. (a) Cumulative coverage of corresponding depth and coverage of corresponding depth in five cultivars. The y axis indicates proportion of C bases in the genome is not less than the depth of the sequence, which accounts for the proportion of all C bases in the genome; x axis indicates read depth. (b) Depth distribution of DNA sequencing. The y axis indicates percentage of the sequenced depth; the axis indicates cumulative sequencing depth and sequencing depth, respectively. (c) The percentage of methylcytosines identified for each sequence context from five apple cultivars.

**Fig. S3.** Global methylome maps of apple cultivars. Density plots of mCs in different sequence contexts (A, CHH; B, CHG and C, CG), CG ratio (D), CGI density (E), transposable elements density (F) and gene density (G) are shown in the maps. Chromosome name is indicated on the outer rim.

**Fig. S4.** Distribution of mCs on the chromosomes of five apple cultivars. Distribution of mCs on each chromosome in NF2, YF3 and YF8 cultivars (a) and RL and SNH cultivars (b). The horizontal axis represents the chromosome, from left to right indicate the starting and ending point of the chromosome. The vertical axis on the left shows the mC density calculated for the window of 10 kb, the blue dot indicates the distribution of mC density on the chromosome. The lines represent mC densities in CG (green), CHG (blue), and CHH (orange) contexts, respectively.

**Fig. S5.** KEGG analysis of DMRs in three comparisons. Differential methylation region in CG and CHG (CHH was not found) were analyzed in NF2/YF3 (a, b), YF3/YF8 (c, d) and RL/SNH (e, f), respectively. a, b, c represent DMRs-associated in genes and b, d, f represent DMRs-associated in promoters. Size of the circle represents gene numbers and the blue color represents the q-value.

**Fig. S6.** Differential gene expression in three comparisons. Volcano plot of differential gene expression in three comparisons: NF2/YF3 (a), YF3/YF8 (b) and RL/SNH (c). Orange spots indicate up-regulated genes and the blue indicate down-regulated genes. (d) Numbers of differentially expressed genes (DEGs) in three comparisons.

**Fig. S7.** Gene Ontology (GO) and KEGG pathway analyses of the DEGs among the three comparisons. (a) Go analyses for NF2/YF3, YF3/YF8 and RL/SNH comparisons. (b) KEGG pathway analyses for NF2/YF3, YF3/YF8 and RL/SNH comparisons.

**Fig. S8.** Validation of DNA methylation results in three comparisons. (a) Validation of the DNA methylation of CG in promoter regions. (b) Validation of the DNA methylation of CG in gene regions. (c) Validation of the DNA methylation of CHG in gene region. The vertical axis represents the value of log_2_ (Fold Change) and the horizontal axis indicates candidate genes. Genes are listed: *F3H* (MD05G1074200), *ANS* (MD07G1010700), *ABC* (MD13G1109600), *MATE* (MD07G1010700), *MYB114* (MD17G1261100), *bHLH* (MD16G1247600). The bars represent standard error.

**Fig. S9.** Validation of the expression level of candidate genes related to the anthocyanin biosynthetic pathway in five cultivars. (a) Validation of the expression level of candidate genes in NF2/YF3. (b) Validation of the expression level of candidate genes in YF3/YF8. (c) Validation of the expression level of candidate genes in RL/SNH. The vertical axis indicates the value of log_2_ (Fold Change) and the horizontal axis represents candidate genes. Genes are listed: *CHS* (MD04G1003300, MD13G1285100), *CHI* (MD01G1167300), *ANS* (MD03G1001100), *UFGT* (MD01G1234400, MD17G1055500), *GST* (MD05G1184300, MD10G1172100, MD17G1272100), *MATE* (MD04G1165300, MD07G1010700), *ABC* (MD13G1109600), *MYB114 (*MD17G1261100). The bars represent standard error, ‘*’ and ‘**’indicate significance difference at P<0.05 and P<0.01, respectively.
